# Maternal healthcare utilization and full immunization coverage among 12–23 months children in Benin: a cross sectional study using population-based data

**DOI:** 10.1186/s13690-021-00554-y

**Published:** 2021-03-16

**Authors:** Eugene Budu, Abdul-Aziz Seidu, Ebenezer Agbaglo, Ebenezer Kwesi Armah-Ansah, Kwamena Sekyi Dickson, Thomas Hormenu, John Elvis Hagan, Collins Adu, Bright Opoku Ahinkorah

**Affiliations:** 1 Department of Population and Health, College of Humanities and Legal Studies, University of Cape Coast, Cape Coast, Ghana; 2 College of Public Health, Medical and Veterinary Sciences, James Cook University, Townsville, Queensland Australia; 3 Department of English, University of Cape Coast, Cape Coast, Ghana; 4 Department of Health, Physical Education, and Recreation, University of Cape Coast, Cape Coast, Ghana; 5 Neurocognition and Action-Biomechanics-Research Group, Faculty of Psychology and Sport Sciences, Bielefeld University, Bielefeld, Germany; 6 Department of Health Promotion and Disability Studies, Kwame Nkrumah University of Science and Technology, Kumasi, Ghana; 7 School of Public Health, Faculty of Health, University of Technology Sydney, Sydney, NSW Australia

**Keywords:** Maternal healthcare utilization, Full immunization coverage, Benin, Public health

## Abstract

**Background:**

Maternal and child health are important issues for global health policy, and the past three decades have seen a significant progress in maternal and child healthcare worldwide. Immunization is a critical, efficient, and cost-effective public health intervention for newborns. However, studies on these health-promoting indicators in low-income and middle-income countries, especially in sub-Sahara Africa are sparse. We investigated the association between maternal healthcare utilization and complete vaccination in the Republic of Benin.

**Methods:**

We analysed data from the 2018 Benin Demographic and Health Survey (BDHS). Specifically, the children’s recode file was used for the study. The outcome variable used was complete vaccination. Number of antenatal care visits, assistance during delivery, and postnatal check-up visits were the key explanatory variables. Bivariate and multilevel logistic regression analyses were carried out. The results were presented as unadjusted odds ratios (uOR) and adjusted odds ratios (aOR), with their corresponding 95% confidence intervals (CIs) signifying their level of precision. Statistical significance was declared at *p* < 0.05.

**Results:**

The prevalence of full immunization coverage in Benin was 85.4%. The likelihood of full immunization was lower among children whose mothers had no antenatal care visits, compared to those whose mothers had 1–3 visits [aOR = 0.11, 95% CI: 0.08–0.15], those who got assistance from Traditional Birth Attendants/other during delivery, compared to those who had assistance from Skilled Birth Attendants/health professionals [aOR = 0.55, 95% CI: 0.40–0.77], and mothers who had no postnatal care check-up visit, compared to those who had postnatal care check-up < 24 h after delivery [aOR = 0.49, 95% CI: 0.36–0.67]. With the covariates, religion, partner’s level of education, parity, wealth quintile, and place of residence also showed significant associations with full immunization.

**Conclusion:**

The study has demonstrated strong association between full immunization and antenatal care, skilled attendance at birth, and postnatal care check-up visit. We found that full immunization decreases among women with no antenatal care visits, those who receive assistance from Traditional Birth Attendants during delivery, and those who do not go for postnatal care visits. To help achieve full immunization, it is prudent that the government of Benin collaborates with international organisations such as WHO and UNICEF to provide education to pregnant women on the importance of immunization after delivery. Such education can be embedded in the antenatal care, delivery and postnatal care services offered to pregnant women during pregnancy, delivery, and after delivery.

**Supplementary Information:**

The online version contains supplementary material available at 10.1186/s13690-021-00554-y.

## Background

Maternal and child health is essential in global health policy, and the past three decades have shown significant progress in maternal and child healthcare worldwide. Evidently, there was a decline in maternal mortality ratio (MMR) globally from 385 to 211 deaths per 100,000 live births from 1990 to 2017 [1–3]. In spite of this trend, close to one million women worldwide have lost their lives during pregnancy, childbirth, or within few weeks after delivery, with women in sub-Saharan Africa being the most vulnerable [4, 5]. This challenge has been due to under-utilization of maternal healthcare services in the sub-region [6]. In Benin, MMR as of 2017 was 397 per 100, 000 live births [7].

It is estimated that about two thirds of neonatal mortality could be prevented through maternal and child health programmes, such as antenatal care (ANC), skilled birth delivery, and postnatal care (PNC) [8, 9]. Concerning PNC, the World Health Organization (WHO) recommends that mothers and newborns receive PNC in health posts for at least 24 h if the delivery took place in a health facility; otherwise, PNC should be within 24 h of birth [5]. Basic care for all newborns according to the WHO should include promoting, supporting early, and exclusive breastfeeding, keeping the baby warm, increasing hand washing, providing hygienic umbilical cord and skin care, as well as identifying conditions requiring additional care and counselling on when to take a newborn to a health facility. Newborns and their mothers should be examined for the danger signs at home visits. At the same time, families should be counselled on identification of these danger signs and the need for prompt care seeking if one or more of them are present [5]. However, PNC seems to be underused in sub-Saharan Africa, causing high rates of neonatal mortality each year [10], and estimated at 1.16 million deaths in the first 28 days of life and 850,000 deaths within the first week of birth [10]. Benin’s neonatal mortality rate stood at 31 deaths per 1000 live births in 2016 [11].

Immunization, a critical, efficient, and cost-effective public health intervention [12–15], is one of the postnatal care services for newborns [5]. Immunization is associated with a lot of health, social, and developmental benefits to nations, communities, households, and individuals [16]. Currently, each year, immunization saves 2 to 3 million children from dying [17]. It is recommended that immunization must be administered right after birth within a specified schedule and time range [18]. The target two of the Sustainable Development Goal (SDG) 3 highlights the significance of improving child health and survival rates, and has led to the improvement of public and private investments in promoting accessible and affordable child health intervention programs, particularly immunization services [19]. This has resulted in immunization of about 116 million children against diphtheria, tetanus, and pertussis (DTP), with a coverage rate of 83% in 2018, up from 72% in 2000 and 20% in 1980 globally [20, 21].

Despite the progress made over the years in terms of immunization, full immunization coverage in SSA has remained undesirable, resulting in poor child health outcomes [22, 23]. Particularly in Benin, previous studies have shown that full immunization coverage increased from 36 to 58.1% between 2012 and 2018 [24, 25]. The current prevalence of complete vaccination in Benin is still low compared to the global prevalence and most countries in SSA. However, the factors that determine full vaccination coverage in Benin is not known. Moreover, Benin records low use of maternal health care services, with about 77% of the population living about 3 miles away from health facilities [26]. Some studies on full childhood immunization have been conducted, focusing on Ethiopia [27–29], Burkina Faso [27], Malawi [30], and Nigeria [31]. Such studies have revealed some associations between full immunization and factors such as age of mother [31], means of transportation to nearby health facility [29], mother’s wealth status [30], and mother’s educational background [27]. In the present study, we assessed the relationship between maternal healthcare utilization and full immunization in Benin, while controlling significant individual and contextual level factors. Findings of the study will enhance information on maternal healthcare utilization, which forms an essential component for full immunization coverage in the country. This data is likely to help reduce child mortality in Benin through planned interventions and re-designing of policies.

## Methods

### Data source and sample

The study used data from the 2018 Benin Demographic and Health Survey (BDHS). Specifically, the birth recode file was used. The survey focuses on essential maternal and child health markers, including immunization [32]. To ensure accuracy of data collection, data collection was done by survey staff who were trainees and were given instructions in standard DHS procedures. These procedures included general interviewing techniques, conducting interviews at the household level, and review of each question and mock interviews between participants. To ensure participants understood the questions being asked, the definitive questionnaires were first prepared in English and subsequently translated by experts into the major local languages at the various data collection points. Interviews were also conducted in local languages. As part of quality assurance, pretest training and field practice of the DHS survey protocol and instruments were done. Field staff were further given training before the actual data collection to ensure that they were able to gain accurate understanding of the data collection instruments. The DHS questions were also standardized, making it possible to do cross-country studies. The surveys employed a two-stage stratified sampling technique, which makes the survey data nationally representative [33]. The first-stage involved a listing of primary sampling units (PSUs) or enumeration areas (EAs) that covered the entire country and usually were obtained from the latest national census. These EAs are also known as the clusters. Each EAs was further subdivided into standard size segments and a sample of predetermined segments were selected randomly with probability proportional to the number of households in each EA. In the second stage, households were systematically selected by surveying the personnel from a list of previously enumerated households in each selected EA segment, and in-person interviews were conducted in selected households to target populations: women aged 15–49, men aged 15–64, and children under 5. In this study, a total of 4156 married and cohabiting women who had complete information on all the variables of interest were considered as the sample size. The dataset can be accessed at https://dhsprogram.com/methodology/survey/survey-display-491.cfm. We relied on the Strengthening the Reporting of Observational Studies in Epidemiology’ (STROBE) statement in conducting this study and writing the manuscript.

### Study variables

#### Outcome variable

The study used complete vaccination as the outcome variable. In this study, complete and full immunisation coverage are used interchangeably. ﻿The information on vaccination coverage was collected from either immunization cards or from mothers’ verbal responses to these questions “Did (NAME) ever receive vaccination against Measles?”, “Did (NAME) ever receive vaccination against Polio?”, “Did (NAME) ever receive vaccination against BCG?”, and “Did (NAME) ever receive vaccination against DPT?”. Responses were “Yes”, “No” and “Don’t Know.” These responses were coded as “No” = 0, “Yes = 1″ and “Don’t Know = 8″. For the purpose of the analysis, only women who provided definite responses (either “Yes” or “No”) were included in the study. ﻿According to the WHO guideline [34], “complete or full immunization” coverage is defined as a child that has received one dose of BCG, three doses of pentavalent, pneumococcal conjugate (PCV), oral polio vaccines (OPV); two doses of Rota virus, and one dose of measles vaccine. We recoded each variable (vaccinations) as “0″ and “1″ for children who didn’t take the recommended doses and those who took respectively. The complete vaccination was obtained by creating a composite variable which comprised all the vaccines administered. To provide a binary outcome, the two responses were coded as follows: “Incomplete” = 0, “Complete = 1″.

#### Independent variables

The study used three indicators as key independent variables. These indicators are number ANC visits, assistance during delivery and the period when postnatal checks were done. The choice of these variables was based on their consideration as the key components of maternal healthcare in the DHS as well as their significant associations with full vaccination in previous studies [35–41]. These variables were generated from the questions, “how many times did you attend ANC during pregnancy?”, “who assisted in the delivery of your baby?” and “when after birth did PNC checks occur?”. Number of ANC visits was coded as ‘0, 1-3 and 4+’. Delivery assistance was coded as ‘Traditional Birth Attendants (TBA)/others and Skilled Birth Attendants (SBA)/health professional. The PNC check-up visits time was coded into ‘No, <24 hours, and >=1 day’.

#### Control variables

Eighteen control variables were considered. These variables were categorised into two broad factors. These factors were individual level (i.e., sex of the child, size of the child at birth, type of delivery, twin status, maternal age, marital status, occupation, mother’s education, partner’s education, religion, ethnicity, parity, frequency of reading newspaper or magazine, frequency of listening to radio, and frequency of watching television), and contextual level factors (i.e., wealth index, place of residence, and region) These variables were considered because of their statistically significant relationships with the full vaccination in previous studies [42–45].

### Statistical analysis

Data were processed and analyzed using Stata version 14.0 with the use of both inferential and descriptive statistics. Prior to the data analysis, data cleaning was done and missing data in the form of blanks. The screening processes explained in the DHS as representing not applicable for the respondent either because the question was not asked in a particular country or because the question was not asked of this respondent due to the flow or skip pattern of the questionnaire were observed for ANC (167 cases), postnatal care (244), partner’s educational level (213), and birth size (43). These missing data were deleted through listwise deletion in order to get respondents with complete cases for the analyses. The analyses were done in three steps. In the first step, descriptive statistics (frequency and percentages) were used to describe the characteristics of the respondents (see Table 1). Next, a bivariate logistic regression analysis was conducted to examine the unadjusted relationship among maternal healthcare utilization, individual, contextual level factors, and full immunization. The results were presented as unadjusted odds ratios (uOR). All the variables that showed statistical significance (*p* < 0.05) were moved to the third step of the analysis. The third step was the use of multilevel models to examine the association between maternal healthcare utilization and full vaccination, while controlling for the individual and contextual level factors. The unit of analysis was married and cohabiting women who had at least one birth at the time of the survey. Five multilevel models (Model 0, I, II, III and IV) were built using only variables which were significant from the bivariate analysis in the second step of the analysis. Model 0 was regarded as the empty model that showed the variations in full immunization without any of the explanatory variables. Statistical significance at this level provides the basis for the use of multilevel models. In Model I, the key independent variables (ANC, assistance during delivery and PNC check-up visits) were included. Model II contained only the individual level variables, Model III had only the contextual level variables, and Model IV had the key independent, individual, and the contextual level variables. To ensure the accuracy of the results, a sensitivity analysis was performed using single women (never married, widowed, separated, and divorced). The results were presented in supplementary Table S1, with adjusted odds ratios (aOR) and their corresponding 95% confidence intervals signifying the level of precision. Statistical significance was declared at *p* < 0.05. For comparing models, the Akaike’s Information Criterion (AIC) and the log likelihood tests were used. The lowest AIC and the highest log likelihood ration were used to determine the best fit model. Sample weight was applied and the survey command (svy) was used to account for the complex sampling design of the survey.

**Table 1:** Full immunization in 12–23 months children in Benin and unadjusted Odds Ratio by explanatory variables (Weighted)

Variable	Frequency	Percentage	Full immunization	uOR	95% CI	
National	4156	100	85.4			
					**Lower**	**Upper**
**ANC visits**						
0	474	11.4	33.1	1		
1–3	1481	35.6	89.5	18.26^***^	14.20	23.47
4+	2201	53.0	93.8	31.39^***^	24.31	40.54
**Delivery assistance**						
By TBA/Others	903	21.7	60.1	1		
By SBA/Health professional	3253	78.3	92.4	8.50^***^	7.07	10.22
**PNC time**						
No	1372	33.0	69.2	1		
< 24 h	2571	61.9	93.3	6.45^***^	5.33	7.82
> =1 day	213	5.1	93.2	6.79^***^	3.97	11.59
**Birth Size**						
Larger than average	1193	28.7	86.5	1		
Average	2365	56.9	85.5	0.96	0.79	1.17
Smaller than average	598	14.4	82.6	0.79	0.60	1.03
**Type of delivery**						
Virginal birth	3978	95.7	85.0	1		
Cesarean section	178	4.3	92.7	2.37^**^	1.34	4.20
**Twin status**						
Single birth	4064	97.8	85.3	1		
Multiple birth	92	2.2	86.4	1.14	0.62	2.11
**Sex of child**						
Male	2086	50.2	86.1	1		
Female	2070	49.8	84.6	0.89	0.75	1.05
**Mother’s age**						
15–19	293	7.0	84.0	1		
20–24	1043	25.1	87.1	1.26	0.88	1.79
25–29	1280	30.8	85.4	1.15	0.81	1.61
30–34	812	19.5	85.1	1.07	0.75	1.53
35–39	504	12.1	86.0	1.22	0.82	1.82
40–44	165	4.0	78.1	0.67	0.42	1.09
45–49	58	1.4	77.5	0.71	0.36	1.41
**Marital status**						
Married	3251	78.2	84.7	1		
Cohabiting	905	21.8	87.7	1.43^**^	1.14	1.79
**Occupational status**						
Not Working	821	19.7	80.4	1		
Working	3335	80.3	86.6	1.48^***^	1.21	1.81
**Wealth quintile**						
Poorest	904	21.8	67.2	1		
Poorer	861	20.7	83.2	2.20^***^	1.76	2.74
Middle	872	21.0	89.6	4.15^***^	3.20	5.37
Richer	802	19.3	92.9	6.53^***^	4.81	8.86
Richest	716	17.2	97.2	16.25^***^	10.41	25.36
**Religion**						
Christianity	2068	49.8	92.0	1		
Islam	1401	33.7	76.9	0.26^***^	0.23	0.34
Other	687	16.5	82.6	0.38^***^	0.30	0.49
**Mother’s education**						
No education	2651	63.8	80.8	1		
Primary	763	18.4	91.3	2.39^***^	1.84	3.10
Secondary/tertiary	742	17.8	95.5	5.62^***^	3.89	8.11
**Partner’s education**						
No education	2245	54.0	78.3	1		
Primary	864	20.8	91.3	2.52^***^	1.97	3.22
Secondary/tertiary	1047	25.2	95.5	5.74^***^	4.26	7.74
**Ethnicity**						
Adja	570	13.7	87.9	1		
Bariba	563	13.7	89.2	1.00	0.70	1.45
Dendi	278	6.7	82.2	0.61^*^	0.41	0.91
Fon	1395	33.6	93.4	1.74^***^	1.26	2.41
Yoa and Lokpa	129	3.1	91.7	1.40	0.72	2.73
Betamaribe	294	7.1	84.9	0.69	0.47	1.03
Peulh	497	12.0	52.9	0.15^***^	0.11	0.20
Yoruba	429	10.3	88.9	0.97	0.67	1.42
**Parity**						
One birth	840	20.2	90.9	1		
Two births	781	18.8	85.6	0.61^**^	0.45	0.83
Three births	718	17.3	97.5	0.69^*^	0.50	0.95
Four or more births	1816	43.7	81.8	0.45^0.35^	0.59	
**Frequency of reading newspaper or magazine**						
Not at all	3917	94.2	84.8	1		
Less than once a week	145	3.5	96.2	5.26^***^	2.15	12.89
At least once a week	94	2.3	93.7	3.44^***^	1.39	8.51
**Frequency of listening to radio**						
Not at all	1830	44.0	80.4	1		
Less than once a week	915	22.0	88.4	1.79^***^	1.42	2.25
At least once a week	1411	34.0	89.8	2.12^***^	1.73	2.60
**Frequency of watching television**						
Not at all	2765	66.5	81.6	1		
Less than once a week	689	16.6	89.5	1.83^***^	1.42	2.36
At least once a week	702	16.9	96.0	5.20^***^	3.59	7.55
**Place of residence**						
Urban	1532	36.9	91.0	1		
Rural	2624	63.1	82.1	0.45^***^	0.37	0.54
**Region**						
Alibori	592	14.3	77.1	1		
Atacora	379	9.1	80.8	1.30	0.95	1.78
Atlantic	410	9.9	93.8	3.61^***^	2.34	5.59
Borgou	545	13.1	69.8	0.66^***^	0.50	0.86
Collines	272	6.5	90.9	2.94^***^	1.94	4.45
Couffo	304	7.3	82.3	1.49^***^	1.04	2.13
Donga	236	5.7	89.9	2.50^***^	1.61	3.88
Littoral	163	3.9	96.3	10.38^***^	4.77	22.61
Mono	187	4.5	94.1	5.55^***^	2.93	10.53
Oueme	360	8.7	96.1	7.25^***^	4.02	13.09
Plateau	268	6.4	83.1	4.11^***^	2.70	6.28
Zou	439	10.6	92.9			

*uOR* unadjusted Odds Ratio, *CI* Confidence Interval 1 = reference category ^*^ *p* < 0.05, ^**^ *p* < 0.01, ^***^ *p* < 0.001

Source: 2018 Benin Demographic and Health Survey

### Ethical approval

The 2018 BDHS report indicated that ethical approval was granted by the ICF Institutional Review Board. Both written and oral Informed consent was sought from all the participants during the data collection exercise including the emancipated adults (i.e those below 16 years). We requested for the dataset on 10th March, 2020 and was granted access. It was downloaded and kept safe from third parties using ‘my lock box’ after permission was granted.

## Results

Table 1 shows results of the prevalence of full immunization coverage in 12–23 months children in Benin and the bivariate association between maternal healthcare services utilization, individual and contextual factors as well as full immunization. The prevalence of full immunization in Benin was 85.4%. The highest prevalence of full immunization coverage was found in children of mothers who had 4+ ANC visits (93.8%), children of mothers who were assisted by SBA/health professionals during delivery (92.4%), and those who had postnatal check-up visits < 24 h after delivery (93.3%). The results of the unadjusted odds ratios of the determinants of full immunization showed variations in all the explanatory variables except birth size, sex of the child, mother’s age and twin status. In the analysis using single women, the results showed significant association between ANC, delivery assistance, PNC time, mother’s age, religion, mother’s educational level, ethnicity, parity, wealth quintile, and full immunization (see Supplementary Table S1).

Table 2 presents results of the multilevel logistic regression analysis on factors associated with full immunization in 12–23 months children in Benin. The final model (Model IV), presents results of the interaction between maternal healthcare utilization variables, individual and contextual level factors, and full immunization coverage. The likelihood of full immunization was lower among children whose mothers had no ANC visits, compared to those whose mothers had 1–3 visits [aOR = 0.11, 95% CI: 0.08–0.15], those who got assistance from TBAs/other during delivery, compared to those who had assistance from SBAs/health professionals [aOR = 0.55, 95% CI: 0.40–0.77], and mothers who had no PNC check-up visit, compared to those who had PNC check-up < 24 h after delivery [aOR = 0.49, 95% CI: 0.36–0.67]. With the covariates, religion, partner’s level of education, parity, wealth quintile, and place of residence also showed significant associations with full immunization. With the random effects results, the complete model (Model IV), which included all the maternal health utilization indicators, individual and contextual level factors in the model, had an AIC of 2443.268 and a log-likelihood ratio of − 1173.6338. This model was considered as the best fit for predicting the occurrence of full immunization among childbearing women. The results of the multilevel analysis among single mothers showed significant association between only ANC and full immunization [aOR = 13.55, 95% CI: 2.77–66.19] (see Supplementary Table S1).

**Table 2:** Mixed effects results on factors associated with full immunization in 12–23 months children in Benin

Variables	Model 0	Model I	Model II	Model III	Model IV
		aOR 95% CI	aOR 95% CI	aOR 95% CI	aOR 95% CI
**ANC visits**					
0		0.09^***^ (0.07–0.13)			0.11^***^ (0.08–0.15)
1–3		1			1
4+		1.42^**^ (1.09–1.85)			1.20 (0.91–1.59)
**Delivery assistance**					
By TBA/Others		0.46^***^ (0.34–0.63)			0.55^***^ (0.40–0.77)
By SBA/Health professional		1			1
**PNC time**					
No		0.47^***^ (0.35–0.62)			0.49^***^ (0.36–0.67)
< 24 h		1			1
> =1 day		1.04 (0.57–1.90)			1.07 (0.58–1.97)
**Type of delivery**					
Virginal birth			1		1
Cesarean section			1.07 (0.55–2.10)		0.56 (0.29–1.08)
**Marital status**					
Married			1		1
Cohabiting			1.13 (0.84–1.53)		1.37 (0.99–1.90)
**Occupational status**					
Not Working			0.71^*^ (0.54–0.92)		0.78 (0.58–1.05)
Working			1		1
**Religion**					
Christianity			1		1
Islam			0.91 (0.61–1.35)		0.95 (0.61–1.47)
Other			057^***^ (0.41–0.79)		0.71^*^ (0.51–1.00)
**Mother’s education**					
No education			1		1
Primary			1.06 (0.77–1.48)		0.99 (0.70–1.40)
Secondary/tertiary			1.70^*^ (1.05–2.76)		1.16 (0.70–1.92)
**Partner’s education**					
No education			1		1
Primary			1.36 (0.99–1.86)		1.06 (0.75–1.49)
Secondary/tertiary			2.16^***^ (1.47–3.18)		1.55^*^ (1.03–2.35)
**Ethnicity**					
Adja			0.69 (0.44–1.09)		1.21 (0.57–2.57)
Bariba			0.89 (0.52–1.53)		1.58 (0.70–3.57)
Dendi			0.48^*^ (0.25–0.93)		0.72 (0.29–1.82)
Fon			1		1
Yoa and Lokpa			1.08 (0.44–2.61)		1.56 (0.48–5.02)
Betamaribe			0.73 (0.42–1.29)		2.05 (0.84–4.98)
Peulh			0.13^***^ (0.08–0.23)		0.70 (0.31–1.59)
Yoruba			0.62 (0.38–1.00)		1.17 (0.64–2.14)
**Parity**					
One birth			1.99^***^ (1.44–2.75)		1.62^**^ (1.14–2.30)
Two births			0.94 (0.71–1.25)		0.89 (0.65–1.21)
Three births			1.17 (0.87–1.58)		1.20 (0.86–1.67)
Four or more births			1		1
**Frequency of reading newspaper or magazine**					
Not at all			1		1
Less than once a week			1.14 (0.42–3.09)		1.14 (041–3.18)
At least once a week			0.62 (0.22–1.77)		0.64 (0.22–1.86)
**Frequency of listening to radio**					
Not at all			1		1
Less than once a week			1.20 (0.90–1.61)		1.06 (0.77–1.46)
At least once a week			1.27 (0.96–1.69)		1.04 (0.77–1.42)
**Frequency of watching television**					
Not at all			1		1
Less than once a week			1.17 (0.84–1.63)		1.04 (0.73–1.50)
At least once a week			2.11^**^ (1.34–3.30)		1.37 (0.82–2.29)
**Wealth quintile**					
Poorest				1	1
Poorer				1.78^***^ (1.36–2.33)	1.14 (0.83–1.55)
Middle				3.29^***^ (2.40–4.52)	1.81^**^ (1.26–2.59)
Richer				3.97^***^ (2.72–5.79)	1.77^*^ (1.15–2.72)
Richest				8.46^***^ (4.74–15.10)	2.44^*^ (1.25–4.77)
**Place of residence**					
Urban				1.61^**^ (1.13–2.28)	1.43^*^ (1.02–2.00)
Rural				1	1
**Region**					
Alibori				1	1
Atacora				1.39 (0.76–2.56)	0.94 (0.45–1.95)
Atlantic				2.14^*^ (1.08–4.27)	0.67 (0.26–1.75)
Borgou				0.65 (0.37–1.14)	1.22 (0.67–2.20)
Collines				2.18^*^ (1.11–4.30)	0.70 (0.29–1.68)
Couffo				1.05 (0.56–1.96)	0.47 (0.16–1.39)
Donga				1.79 (0.85–3.78)	1.52 (0.66–3.51)
Littoral				1.96 (0.69–5.56)	0.74 (0.23–2.44)
Mono				4.78^***^ (2.00–11.39)	1.52 (0.45–5.13)
Oueme				3.76^**^ (1.67–8.46)	1.29 (0.46–3.66)
Plateau				0.92 (0.48–1.78)	0.41 (0.16–1.04)
Zou				2.59^**^ (1.33–5.04)	0.94 (0.36–2.43)
**Random effect result**					
PSU variance (95% CI)	2.15 (1.63–2.82)	0.72 (0.47–1.11)	1.12 (0.80–1.56)	0.99 (0.70–1.40)	0.56 (0.34–0.92
ICC	0.3949477	0.1803875	0.2531715	0.231711	0.145173
LR Test	Chi-square = 416.61, *p* = 0.0000	Chi-square = 55.42, p = 0.0000	Chi-square = 145.95, p = 0.0000	Chi-square = 134.07, p = 0.0000	Chi-square = 35.60, p = 0.0000
Wald chi-square		556.67^***^	266.05^***^	214.86^***^	594.80^***^
Model fitness					
Log-likelihood	− 1565.0389	− 1241.965	− 1417.3607	− 1453.5992	−1173.6338
AIC	3134.078	2497.93	2888.721	2943.198	2443.268
N	4156	4156	4156	4156	4156
Number of clusters	547	547	547	547	547

*aOR* adjusted Odds Ratio, 95% confidence intervals in brackets, ^*^ *p* < 0.05, ^**^ *p* < 0.01, ^***^ *p* < 0.001;1 = reference category

Source: 2018 Benin Demographic and Health Survey

## Discussion

The present study investigated the association between maternal healthcare utilization (ANC, skilled birth attendants, and PNC) and full immunization of children aged 12–23 months in Benin. The prevalence of full immunization recorded was 85.4%. This result on the prevalence of full immunization coverage is higher than what was recorded by Efendi et al. [46] in Indonesia (37.4%), Adedire et al. [31] in Atakumosa-west district of Nigeria (58%), Yismaw et al. [29] in Gondar city administration of Northwest Ethiopia (75.5%), Ntenda [30] in Malawi (72%), Aalemi et al. [47] in Afghanistan (48%), and Noh et al. [48] in Sindh, Pakistan (51.3%). This high prevalence of full immunization recorded in Benin could be explained in the light of certain developments. Paul et al. [49], for instance, noted an attempt by the Benin government to invest in activities such as the use of motorbikes to facilitate immunization. The use of motorbikes will help provide vaccination to children who live in hard-to-reach areas where other means of transport such as vehicles are not possible. The high prevalence of full immunization observed is an indication of improvement of child healthcare services in Benin. This finding could be attributed to the introduction of the 13-valent pneumococcal conjugated vaccine (PCV13), in a single-dose vial, into its Expanded Programme for Immunisation (EPI) with support from Gavi, WHO, UNICEF (United Nations International Children’s Emergency Fund) and the Agence de Médecine Préventive bureau Afrique (AMP) [50]. Notwithstanding, the 14.6% women whose children did not receive full immunization implies that the government of Benin in collaboration with WHO and UNICEF should enhance the EPI programme through education and sensitization. 

Women who had no ANC visits were less likely to record full immunization of their children, compared to those who had 1–3 ANC visits. This finding confirms outcome of studies in Afghanistan [47], Osun State of Nigeria [31], Bangladesh [51], Uganda [52], Sindh, Pakistan [48], and Indonesia [46]. In explaining this association, Dixit, Dwivedi, and Ram [53] noted that women who make regular ANC visits easily familiarize themselves with healthcare services such as immunization programmes. Efendi et al. [46] similarly advanced that during antenatal care programs, women are sensitized on issues relating to childcare, including the importance of immunization to children which may account for full immunization. The positive association between ANC and full immunization coverage calls for the need for the Government of Benin, through the Ministry of Health to encourage more pregnant women to access ANC services. During ANC, health workers should also provide adequate information to pregnant women about the importance of immunization after delivery.

Other results show that children’s whose mothers had no PNC check-up had lower likelihood of having full immunization, compared to those whose mothers had PNC check-up < 24 h after delivery. This finding agrees with the findings of Ntenda [30], Aregawi et al. [27], and Mutua et al. [54] in Malawi, Ethiopia, and Kenya respectively. Ntenda [30], for instance, found that children whose mothers had PNC check-up within the last two months prior to the survey had higher chances of having complete immunization. Aregawi et al. [27] similarly revealed a strong association between lack of PNC check-up and incomplete vaccination. A study by Yeni et al. [55] in Ethiopia also revealed that children whose mothers had no PNC visits had higher likelihood of defaulting complete immunization, compared to those whose mothers had PNC visits. In explaining this association, Ntenda [30] revealed that attendance to PNC check-up exposes women to vital information from health workers, including information on the need for full vaccination. This finding implies the need to enhance postnatal care services given to women during postnatal care.

Furthermore, children whose mothers got assistance from TBAs during birth were less likely to access full immunization, as compared to children whose mothers were assisted by SBA/others such as family members. A study by Efendi et al. [46] reported a similar finding in the context of Indonesia. Ahinkorah et al. [56] similarly noted that mothers who were assisted by skilled birth assistants recorded higher likelihoods of seeing the immunization of their children to completion. As noted by Efendi et al. [46], this association is because skilled birth assistants usually educate their clients on postnatal programmes such as immunization. With the acquisition of such information given, such mothers become aware of the need for complete immunization, which encourages them to ensure that their children get access to complete immunization. Women who are assisted by TBAs will lack this information and hence will be less likely to immunize their children. This finding calls for public education on the importance of skilled birth attendance. As part of this education, TBAs should also be educated to provide pregnant women whom they assist during delivery on the importance of childhood vaccination. Such education can be provided by the Government of Benin and other non-governmental organisations with the support of WHO and UNICEF. This call can go a long way to help support the USAID’s Advancing Newborn, Child, and Reproductive Health (ANCRE) Programme which seeks to increase access to quality maternal and child healthcare services.

### Strengths and limitations

The main strength of this study is the use of nationally representative data which makes it possible for the findings to be generalized. The methods employed in sampling and data collection also support the accuracy of the data. Despite these methodological strengths, data on the independent factors and the outcome variable (vaccination) were based on self-reports. Hence, the possibility of recall bias may occur since some mothers (those whose children’s immunization cards were not available) gave retrospective accounts on questions related immunization. Again, the study design was cross-sectional, therefore, results should only be interpreted in terms of associations but not causality. The timing of the survey questions differ. For instance, whiles questions on the individual and contextual factors were in reference to events that occurred during the survey, questions on vaccination may require recall of the time when the child was born.

## Conclusion

The study has demonstrated strong association between ANC, skilled attendance at birth, and PNC and full immunization coverage. We found that full immunization decreases among women with no ANC visits, those who receive assistance from TBAs during delivery and those who do not go for postnatal care visits. To help achieve full immunization, it is prudent that the government of Benin collaborates with international organisations such as WHO and UNICEF to provide education to pregnant women on the importance of immunization after delivery. Such education can be embedded in the ANC, delivery, and PNC services offered to pregnant women during pregnancy, delivery and after delivery.

## Supplementary Information

**Additional file 1: Table S1.** Multilevel analysis among single mothers on maternal healthcare utilization and full immunization coverage among 12–23 months.

## Data Availability

The dataset can be accessed at https:// https://dhsprogram.com/data/dataset/

## Abbreviations

uOR: Unadjusted Odds Ratio
aOR: Adjusted Odds Ratio
CI: Confidence Interval
BDHS: Benin Demographic and Health Survey
VIF: Variance Inflation Factor
SDG: Sustainable Development Goal
ANC: Antenatal Care
PNC: Postnatal Care
MMR: Maternal mortality ratio
SSA: Sub-Saharan Africa
WHO: World Health Organization
PCV: Pneumococcal conjugate
OPV: oral polio vaccine
