# Supplementary material for: Maternal healthcare utilization and full immunization coverage among 12–23 months children in Benin: a cross sectional study using population-based data
Source: Arch Public Health. 2021 Mar 16;79:34. doi: 10.1186/s13690-021-00554-y (PMC7962345; doi:10.1186/s13690-021-00554-y)
Supplement: Supplementary file 1 — Additional file 1: Table S1. Multilevel analysis among single mothers on maternal healthcare utilization and full immunization coverage among 12–23 months. [file 13690_2021_554_MOESM1_ESM.docx]

Table S1: Multilevel analysis among single mothers on **maternal healthcare utilization and full immunization coverage among 12-23 months**

| **Variables** | **cOR** | **aOR** |
| --- | --- | --- |
| **ANC** |  |  |
| 0 | 1 | 1 |
| 1-3 | 12.90^***^ (4.19-39.74) | 13.55^**^ (2.77-66.19) |
| 4+ | 11.53^***^ (4.32-30.77) | 4.43 (0.89-22.03) |
| **Delivery assistance** |  |  |
| By TBA/Others | 1 | 1 |
| By SBA/Health professionals | 3.69^**^ (1.55-8.75) | 0.59 (0.11-3.3) |
| **PNC time** |  |  |
| No | 1 | 1 |
| <24 hours | 4.59^***^ (1.92-11.00) | 0.43 (0.95-15.74) |
| >=1 day | 3.08 (0.38-25.30) | 1.44 (0.11-19.19) |
| **Birth size** |  |  |
| Larger than average | 1 | - |
| Average | 0.52 (0.19-1.47) | - |
| Smaller than average | 0.49 (0.13-1.80) | - |
| **Type of delivery** |  |  |
| Virginal birth | 1 | - |
| Caesarean section | 1.76 (0.22-13.96) | - |
| **Twin status** |  |  |
| Single birth | 1 | - |
| Multiple birth | 1.48 (0.98-1.67) | - |
| **Sex of child** |  |  |
| Male | 1 | - |
| Female | 1.09 (0.49-2.39) | - |
| **Mother’s age** |  |  |
| 15-19 | 1 | 1 |
| 20-24 | 0.32 (0.09-1.16) | 0.21 (0.04-1.27) |
| 25-29 | 1.28 (0.21-8.03) | 2.13 (0.15-30.86) |
| 30-34 | 0.23 (0.05-1.05) | 0.22 (0.01-5.19) |
| 35-39 | 0.63 (0.10-4.02) | 0.73 (0.03-16.57) |
| 40-44 | 0.05^**^ (0.01-0.40) | 0.07 (0.00-8.42) |
| 45-49 | 1.00 | 1 |
| **Marital status** |  |  |
| Never married | 1 | - |
| Widowed/separated/ divorced | 0.63 (0.28-1.38) | - |
| **Occupational status** |  |  |
| Not working | 1 | - |
| Working | 0.80 (0.31-2.08) | - |
| **Religion** |  |  |
| Christian | 1 | 1 |
| Islam | 0.83 (0.30-2.31) | 3.20 (0.66-15.50) |
| Other | 0.23^**^ (0.09-0.61) | 0.51 (0.08-3.51) |
| **Mother’s educational level** |  |  |
| No education | 1 | 1 |
| Primary | 6.16^*^ (1.38-27.52) | 3.15 (0.65-15.39) |
| Secondary/tertiary | 3.49^*^ (1.34-9.12) | 0.50 (0.08-3.01) |
| **Ethnicity** |  |  |
| Adja | 1 | 1 |
| Bariba | 10.00^*^ (1.07-93.85) | 7.92 (0.33-188.60) |
| Dendi | 4.00 (0.41-39.17 ) | 1.39 (0.03-61.98) |
| Fon | 7.25^**^ (1.74-30.21) | 9.54 (0.92-99.30) |
| Yoa and Lakpa | 2.33 (0.23-24.02) | 0.23 (0.02-2.87) |
| Betamaribe | 1.30 (3.80-4.45) | 2.51 (0.16-40.20) |
| Peulh | 0.58 (0.12-2.87) | 0.24 (0.01-9.36) |
| Yoruba | 5.33 (0.92-30.82) | 4.63 (0.48-44.36) |
| **Parity** |  |  |
| One birth | 1 | 1 |
| Two births | 0.34^*^ (0.12-0.92) | 0.16^**^ (0.04-0.63) |
| Three births | 0.79 (0.16-3.87) | 0.99 (0.10-9.87) |
| Four or more births | 0.47 (0.18-1.27) | 0.46 (0.03-6.28) |
| **Frequency of reading newspaper/magazine** |  |  |
| Not at all | 1 | - |
| Less than once a week | 0.54 (0.15-2.02) | - |
| At least once a week | 0.93 (0.11-7.77) | - |
| **Frequency of listening radio** |  |  |
| Not at all | 1 | - |
| Less than once a week | 1.46 (0.50-4.24) | - |
| At least once a week | 1.23 (0.49-3.05) | - |
| **Frequency of watching television** |  |  |
| Not at all | 1 | - |
| Less than once a week | 1.01 (0.36-2.88) | - |
| At least once a week | 3.55 (0.80-15.73) | - |
| **Wealth quintile** |  |  |
| Poorest | 1 | 1 |
| Poorer | 1.17 (0.43-3.16) | 0.41 (0.08-2.15) |
| Middle | 3.50 (0.90-13.65) | 1.38 (0.14-14.05) |
| Richer | 4.23^*^ (1.09-16.41) | 1.06 (0.14-8.03) |
| Richest | 4.07^*^ (1.05-15.80) | 1.18 (0.12-11.27) |
| **Place of residence** |  |  |
| Urban | 1 | - |
| Rural | 0.58 (0.26-1.31) | - |
| **Region** |  |  |
| Alibori | 1 | - |
| Atacora | 1.23 (0.28-5.36) | - |
| Atlantic | 0.90 (0.19-4.32) | - |
| Borgou | 0.79 (0.16-3.81)) | - |
| Collines | 5.44 (0.52-56.99) | - |
| Couffo | 0.47 (0.06-3.57) | - |
| Danga | 1.88 (0.17-20.72) | - |
| Littoral | 1.00 | - |
| Mono | 1.31 (0.12-15.01) | - |
| Oueme | 4.13 (0.39-43.62) | - |
| Plateau | 0.66 (0.09-4.86) | - |
| Zou | 1.00 | - |
